# Effects of providing free hearing aids on multiple health outcomes among middle-aged and older adults with hearing loss in rural China: a randomized controlled trial

**DOI:** 10.1186/s12916-022-02323-2

**Published:** 2022-04-19

**Authors:** Xin Ye, Dawei Zhu, Siyuan Chen, Xuefeng Shi, Rui Gong, Juncheng Wang, Huibin Zuo, Ping He

**Affiliations:** 1 China Center for Health Development Studies, Peking University, 38 Xue Yuan Road, Haidian District, Beijing, 100191 China; 2 School of Management, Beijing University of Chinese Medicine, 11 North 3rd Ring Road East, Chaoyang District, Beijing, 100029 China; 3 China Rehabilitation Research Center for Hearing and Speech Impairment, No. 8 Huixinli A, Chaoyang District, Beijing, 100029 China; 4 Linyi Center for Disease Control and Prevention, 3 Beijing Road, Beicheng New District, Linyi City, 276007 Shandong China

**Keywords:** Hearing aids, Quality of life, Social activities, Social network, China

## Abstract

**Background:**

Hearing loss has been associated with serious health problems around the globe. Previous studies have found the positive effects of fitting hearing aids on health, but few studies were conducted in developing countries. The aim of this study is to examine the effects of hearing aids on multiple health outcomes among middle-aged and older adults with hearing loss in rural China.

**Methods:**

In this randomized controlled trial (RCT), participants aged 45 and above were randomly assigned to the treatment group prescribing with hearing aids or to the control group with no intervention. Trial outcomes for 385 participants were collected during the 20-month follow-up study. Using the difference-in-difference approach, our primary outcomes were hearing-related quality of life (QoL) and generic QoL.

**Results:**

The intervention led to improvements in hearing-related QoL, presenting as the reduction in Hearing Handicap Inventory for the Elderly Screening Version (HHIE-S) scores (interaction coefficient = − 2.86, *p* = 0.005), HHIE-S-Emotional scores (interaction coefficient = − 1.42, *p* = 0.029), and HHIE-S-Situational scores (interaction coefficient = − 1.43, *p* = 0.007). The intervention was also effective in alleviating the increase in depressive symptoms (interaction coefficient = − 0.14, *p* = 0.042). Subgroup analysis revealed that the effects were only shown among people with social activities or active social networks.

**Conclusions:**

Our study is the first RCT in China to measure the health effects and heterogeneity of hearing aid interventions. Wearing hearing aids can help improve hearing-related QoL and alleviate the increase in depressive symptoms. The intervention is expected to be applicable to similar settings in other developing countries to combat hearing-related health problems.

**Trial registration:**

Chinese Clinical Trial Registry, ChiCTR1900024739. Registered on 26 July 2019.

## Background

Hearing loss is the most common sensory dysfunction in human beings and is also a common health issue around the globe [1]. The World Health Organization (WHO) reported that 466 million people suffered from moderate or above hearing loss globally in 2020, accounting for 6.1% of the total population [2]. The risk of hearing loss increases with age. It is said that more than 90% of hearing loss is related to aging, most of which are irreversible [3]. Therefore, the prevalence of hearing loss in older adults is much higher than that in other groups [4]. According to the WHO estimates, in 2018, about 1/3 of the older adults over 65 years old suffered from moderate or above hearing loss worldwide [2]. A population-based hearing epidemiological study conducted in Jilin, Guangdong, Gansu, and Shaanxi provinces of China in 2015 found that the prevalence of hearing loss in older adults over 60 years old was 55.31%, and the prevalence of hearing disability was up to 34.64% [5]. With the aggravation of global aging, hearing loss will become a more urgent public health problem [6].

Hearing loss affects people’s quality of life (QoL) widely [7]. Hearing loss is related to worse physical health, manifesting as more chronic diseases and impaired physical function [3]. People with hearing loss are often isolated from friends and family due to reduced communication skills and increased communication difficulties, which further leads them to restricted social networks and may exacerbate other diseases such as depression and dementia [8, 9]. In addition, hearing loss reduces the individual’s cognitive ability and subjective well-being [10] and leads to emotional loneliness, sadness, despair, helplessness, and negative self-image [11], which may aggravate the decline of mental function [12]. Therefore, untreated hearing loss may have considerable negative social, psychological, cognitive, and health effects on individuals. The 2015 Global Burden of Disease (GBD) study further revealed that hearing loss has become the second chronic disease threatening human health, bringing a heavy social and economic burden [13].

Hearing aid use is currently the most common intervention for age-related hearing loss in adults [14]. Research from observational studies, quasi-experimental studies, and randomized controlled trials (RCTs) have consistently revealed that hearing aid use can improve the hearing function and overall condition of people with hearing loss [15]. However, evidence on the effects of hearing aids on QoL is limited. Hearing aid use is generally considered to improve hearing-related QoL measured by the Hearing Handicap Inventory for the Elderly (HHIE) [16], while there are debates about whether hearing aid use improves generic QoL. Some contended that hearing aids significantly reduced the risk of anxiety and depression, but there was no obvious improvement in mobility, self-care, daily activities, and pain/discomfort in the EuroQoL 5-Dimension (EQ-5D) [17, 18]. Others believed that EQ-5D or the 36-item Short-Form Health Survey (SF-36) did not significantly improve after wearing hearing aids [19, 20].

The results on the effects of hearing aids on broader health outcomes such as mental health, physical health, cognitive function, and social engagement are also inconsistent to some degree. On the one hand, studies found that those using hearing aids had no significant improvement in mental health, cognitive function, social participation, or quality of life compared with the non-users [21–23]. On the other hand, some believed that hearing aid users had a significant decline in perceptions of loneliness, improved social skills, better self-care and daily activities, and better general health [24, 25]. One epidemiological study using data from the National Health and Nutrition Examination Survey (NHANES) found that hearing aid use is independently associated with reduced odds of major depressive disorder and depressive symptoms [11]. In addition, improvements in social engagement, mental health, and cognitive processing has been reported in those using hearing aids [16, 21].

Due to the concealed and progressive nature of age-related hearing loss, individuals especially older adults do not much pay attention to it. Besides, the high costs of hearing aids hinder their use [26]. A systematic review showed that 83% of people with hearing loss did not use hearing aids [27]. According to community-based surveys conducted in developed countries and regions, the proportion of hearing-impaired older adults using hearing aids was about 10–20% [28]. In low- and middle-income countries where healthcare resources are constrained, hearing aid use is lower among hearing-impaired people. Studies in Jilin, Guangdong, Shaanxi, and Gansu provinces of China found that only 6.5% of the older adults with hearing loss had hearing aids [29]. In view of the benefits and low prevalence of hearing aids in low- and middle-income countries, the health effects of hearing aids may be more pronounced. Therefore, this study explores the effects of the hearing aid rehabilitation intervention on QoL and other health outcomes in rural China by providing free hearing aids for middle-aged and older adults with hearing loss.

Based on previous literature, we hypothesized that (1) the hearing aid intervention led to improvements in hearing-related QoL and generic QoL; (2) the intervention reduced the probability of having chronic diseases, impaired ADLs or IADLs, and depressive symptoms; and (3) the effects were different between people with and without social activities or active social network. Our study is novel in that it focuses on a rural sample, while previous studies are not confined to rural communities. Secondly, it includes a diverse set of assessments including evaluations of hearing, depressive symptoms, QoL, ADL/IADL, and social functioning, as existing studies often do not account for all these various factors. In addition, we performed subgroup analysis by social activities and social networks to look at the effect modification, which has never been done before. Our work is of value to a broad audience as it provides the latest evidence in areas of medical and health advances for promoting hearing rehabilitation and achieving healthy aging in developing countries.

## Methods

### Study design and participants

Our trial is a two-arm, randomized controlled trial (RCT) conducted in Linyi City, Shandong Province, China. It aims to improve the QoL of middle-aged and older adults with hearing loss by providing free hearing aids to them. Audiometric testing of pure-tone audiometry (PTA) at the thresholds of 0.5, 1, 2, and 4 kHz was used to assess the degree of hearing loss. PTA was assessed by the study audiologist in an independent audiometry room (background noise ≤ 40 dB) by presenting a pure tone to the ear through an earphone and measuring the lowest intensity in dB at which this tone is perceived 50% of the time.

The inclusion criteria are (1) aged 45 or above, (2) clinically diagnosed with moderate or above hearing loss (defined as a PTA of the four frequencies > 40 dB in the better hearing ear [30]), (3) no hearing aids are currently used, and (4) in the local area during the study. The exclusion criteria are (1) inability to read or write; (2) cognitive, mental, language, or movement disability which was assessed by clinical evaluation and judgment as well as individually administered standardized tests; (3) ever using a hearing aid in the past year; (4) unwilling to wear hearing aids daily; (5) medical contraindication to the use of hearing aids (e.g., otorrhea); and (6) incurable conductive hearing loss, e.g., airbone space adjacent to two or more frequencies > 15 dB. The number of those allocated in the treatment group versus that in the control group was 1:1.5 due to the limited treatment availability.

Of the total sample, 29.61% had a moderate hearing loss, 37.40% had a severe hearing loss, and 32.99% had a profound hearing loss (Table 1). A baseline survey was conducted in July 2019. Those in the treatment group were prescribed with hearing aids (unilateral, behind the ear, with standard button batteries), after which they received real ear measurements to ensure that the hearing aids met the speech communication needs and the hearing loss of the participants. Those in the control group received no interventions. The basic information and health conditions of 385 samples were collected.

**Table 1:** Baseline characteristics and the significance of differences between the treatment and control groups among hearing-impaired middle-aged and older adults

Characteristics	Total sample (***n*** = 385)		Treatment group (***n*** = 150)		Control group (***n*** = 235)		***p*** value
Age (years), mean (SD)	68.50 (0.53)		68.22 (0.94)		68.68 (0.62)		0.670
Sex, *N* (%)							0.746
Male	271	70.39	107	71.33	164	69.79	
Female	114	29.61	43	28.67	71	30.21	
Hukou types, *N* (%)							0.167
Rural	360	93.51	137	91.33	223	94.89	
Urban	25	6.49	13	8.67	12	5.11	
Education, *N* (%)							0.173
Illiterate	179	46.49	65	43.33	114	48.51	
Primary school	130	33.77	59	39.34	71	30.21	
Middle school or above	76	19.74	26	17.33	50	21.28	
Marital status, *N* (%)							0.010
Alone	89	23.12	45	30.00	44	18.72	
Married/partnered	296	76.88	105	70.00	191	81.28	
Work types, *N* (%)							0.518
Non-agriculture	24	6.23	11	7.33	13	5.53	
Agriculture	190	49.35	69	46.00	121	51.49	
Unemployed	171	44.42	70	46.67	101	42.98	
Life sources, *N* (%)							0.129
Only on one’s own	54	14.03	16	10.67	38	16.17	
Have support from others	331	85.97	134	89.33	197	83.83	
Health insurance, *N* (%)							0.125
No	15	3.90	3	2.00	12	5.11	
Yes	370	96.10	147	98.00	223	94.89	
Pure-tone average, *N* (%)							0.990
Moderate (40 dB < PTA ≤ 60 dB)	114	29.61	45	30.00	69	29.36	
Severe (60 dB < PTA ≤ 80 dB)	144	37.40	56	37.33	88	37.45	
Profound (PTA > 80 dB)	127	32.99	49	32.67	78	33.19	
Smoking status, *N* (%)							0.381
Smoke now	123	31.95	43	28.67	80	34.04	
Ever smoke	64	16.62	29	19.33	35	14.90	
Never smoke	198	51.43	78	52.00	120	51.06	
Drinking status, *N* (%)							0.819
Drink now	112	29.09	43	28.67	69	29.36	
Ever drink	71	18.44	30	20.00	41	17.45	
Never drink	202	52.47	77	51.33	125	53.19	
Social activities, *N* (%)							0.993
No	100	25.97	39	26.00	61	25.96	
At least one	285	74.03	111	74.00	174	74.04	
Social network, *N* (%)							0.577
Restricted	101	26.23	37	24.67	64	27.23	
Active	284	73.77	113	75.33	171	72.77	
**Primary outcomes**							
HHIE-S, mean (SD)	31.65	0.39	33.49	0.45	30.47	0.56	< 0.001
HHIE-S-Emotional, mean (SD)	14.01	0.24	15.00	0.30	13.37	0.34	0.001
HHIE-S-Situational, mean (SD)	17.64	0.20	18.49	0.22	17.10	0.29	< 0.001
SF-12-Physical, mean (SD)	40.75	0.43	40.68	0.70	40.80	0.54	0.887
SF-12-Mental, mean (SD)	40.01	0.49	40.99	0.80	39.39	0.62	0.112
EQ-5D-5L, mean (SD)	0.76	0.01	0.75	0.02	0.77	0.01	0.611
**Secondary outcomes**							
Chronic diseases, *N* (%)							0.114
No	101	26.23	46	30.67	55	23.40	
Yes	284	73.77	104	69.33	180	76.60	
ADLs, *N* (%)							0.315
Unimpaired	295	76.62	119	79.33	176	74.89	
Impaired	90	23.38	31	20.67	59	25.11	
IADLs, *N* (%)							0.076
Unimpaired	123	31.95	40	26.67	83	35.32	
Impaired	262	68.05	110	73.33	152	64.68	
Depressive symptoms, *N* (%)							0.453
No	245	63.64	92	61.33	153	65.11	
Yes	140	36.36	58	38.67	82	34.89	
Cognition scores measured by MMSE, mean (SD)	16.93	0.45	17.07	0.75	16.83	0.56	0.796

Twelve months after the intervention, 72.73% of the respondents in the treatment group reported still using hearing aids during the past 2 weeks, of which 27.97% reported using hearing aids less than 4 h a day, 20.28% used hearing aids 4–8 h a day, and 24.48% used hearing aids more than 8 h a day. Hearing aid adjustment, repairment, and reinstruction in the use of hearing aids were provided free of charge to all subjects in the treatment group, which increased the subjects’ compliance with hearing aid use.

**Fig. 1:**
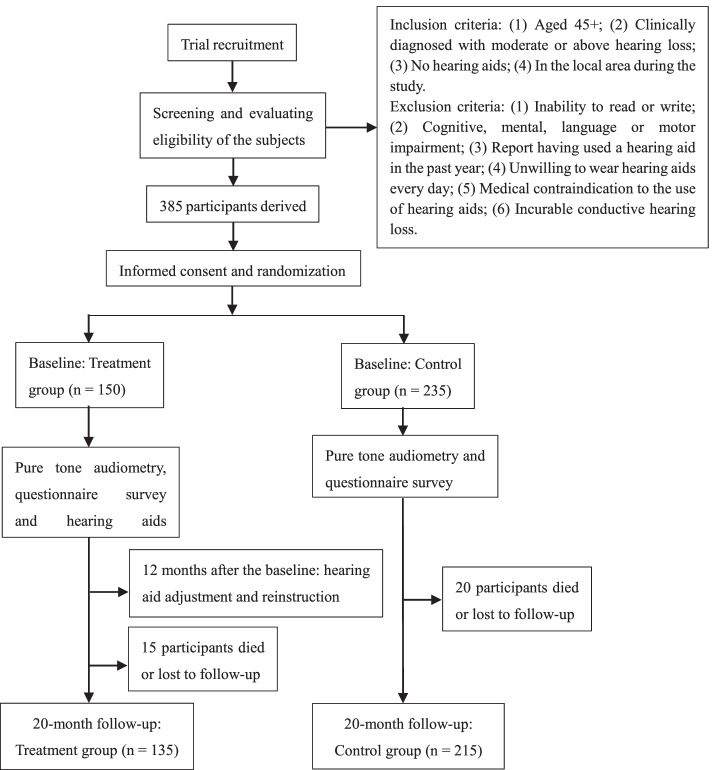
Trial flow diagram for the enrollment and progress of the sample through study

A follow-up survey was conducted 20 months after baseline. A total of 350 samples were followed, and their QoL, physical function, mental health, cognition, and healthcare utilization were tracked. 73.96% of the respondents in the treatment group reported still using hearing aids during the past 2 weeks, of which 30.21% reported using hearing aids less than 4 h a day, 11.46% used hearing aids 4–8 h a day, and 32.29% used hearing aids more than 8 h a day. Figure 1 shows the trial flow diagram for the enrollment and progress of the sample through the study.

As we focus on the effect of hearing aids for those with age-related hearing loss, and due to the concealed and progressive nature of age-related hearing loss, the proportion of adults aged below 45 years old with hearing loss was quite small [31]. Besides, hearing loss is often neglected by middle-aged and older adults, and the lack of hearing aid use is more challenging in this population. To ensure the homogeneity of the target population, we only took into account the participants aged 45 or above. For the inclusion criteria, “some degree of hearing loss” in the protocol actually referred to “moderate or above hearing loss.” More details of our trial design, intervention, and outcomes can be seen in our trial protocol [28].

### Primary outcomes

Primary outcomes are hearing-related QoL and generic QoL. We measured all outcomes at baseline and 20-month follow-up.

1. Hearing-related QoL: The Chinese version of the Hearing Handicap Inventory for the Elderly Screening Version (HHIE-S) is adopted to measure hearing-related QoL. HHIE-S has 10 questions, including 5 emotional questions such as “Do you feel handicapped by a hearing problem?” and 5 situational questions such as “Does a hearing problem cause you difficulty when attending a party?” Subjects are required to answer “yes,” “sometimes,” or “no” according to their own real situation, and the corresponding scores were 4, 2, and 0, respectively. The total score is 0–40 points. A higher score denotes worse hearing-related QoL. A total score of more than 8 is generally considered to represent having hearing loss. The Chinese version of HHIE-S has been proved to be feasible, reliable, and of validity in the measurement of hearing-related QoL in China [32].
2. Generic QoL: a. The 12-item Short-Form Health Survey (SF-12) is used to summarize generic QoL. One of the questions is “Does your health now limit you in climbing several flights of stairs?” It describes QoL from eight dimensions, including physical function, role limitation caused by physical problems, body pain, physical health awareness, social function, role limitation caused by emotional problems, emotional health, and mental health awareness [33]. Among them, the first four items can be added together to produce the SF-12 physical standard value, and the last four items can be added up to derive the SF-12 mental standard value.
3. Generic QoL: b. The EuroQoL 5-Dimension 5-Level (EQ-5D-5L) is adopted to measure generic QoL in terms of mobility, self-care, daily living, pain/discomfort, and anxiety/depression [34]. For example, it asks subjects whether they have difficulty walking. Subjects select the most suitable option among the five levels of “no difficulty,” “a little difficulty,” “moderate difficulty,” “severe difficulty,” and “unable to proceed/have very serious difficulty” in each question according to their health status.

### Secondary outcomes

Secondary outcomes include chronic diseases, activities of daily living (ADLs), instrumental activities of daily living (IADLs), depressive symptoms, and cognitive function. We measured all outcomes at baseline and 20-month follow-up.

1. Chronic diseases: Subjects are asked whether they have at least one chronic disease that has been diagnosed by a doctor, including hypertension, dyslipidemia, diabetes, cancer, chronic lung diseases, liver diseases, heart diseases, stroke, kidney diseases, stomach diseases, digestive system diseases, mental health problems, diseases associated with memory (e.g., Alzheimer’s, brain atrophy, Parkinson’s), arthritis, rheumatism, or asthma.
2. Activities of daily living (ADLs): ADLs are used to measure subjects’ physical functioning. Impaired ADLs are defined as the subject having difficulty or inability to perform any of the activities of daily living, including dressing, bathing, eating, getting up or out of bed, going to the toilet, or controlling urine and feces [35].
3. Instrumental Activities of Daily Living (IADLs): IADLs are defined as whether the subject is having difficulty or inability to perform any of the instrumental activities of daily living, including doing housework, cooking, shopping, making phone calls, taking medications, or managing finances [35].
4. Depressive symptoms: Depressive symptoms of the subjects are detected by using the 10-Item Center for the Epidemiological Studies of Depression Short Form (CES-D-10) [36]. The subjects rate how often each emotion has occurred in the past week on a 4-point scale, ranging from 0 (“none”) to 3 (“most of the time”). Individuals with depressive symptoms scored ≥ 10 points out of a CES-D-10 total score of 0–30.
5. Cognitive function: The subjects’ mental state and cognitive function deficit are tested by the Mini-Mental State Examination (MMSE) [37]. The scale includes the following seven aspects: time orientation, place orientation, immediate memory, attention and computation, delayed memory, language, and visuospatial. There are 30 items in total, with 1 mark for each correct answer and 0 mark for each wrong or unknown answer. The total score range of the scale is 0–30.

### Social activities

Subjects were asked whether they participated in one or more social activities last month, such as socializing with friends; playing mahjong, chess, or cards; going to the community room; offering to help a relative, friend, or neighbor who did not live together; dancing, exercising, and playing Qigong; joining in activities organized by associations; participating in volunteer activities or charitable activities; caring for the sick or disabled who did not live together; attending school or training programs; speculating in stocks, funds, or other financial securities; and surfing the Internet.

### Social network

The Lubben Social Network Scale (LSNS) is a brief instrument used to evaluate the size, closeness, and frequency of contacts of a subject’s perceived social network (covering both kin/family and nonrelated individuals) [38]. The scale consists of an equally weighted sum of 10 items, including two dimensions of family network and friend network, with a score of 0–5 for each question and a total score of 0–50. A higher score indicates a more active social network, and a score less than 20 indicates that individuals are isolated and their social networks are severely restricted [39].

### Statistical analysis

*T*-test for continuous variables and Pearson’s chi-square test for categorical variables were used to examine the extent to which there was a balance between characteristics of the intervention and control groups at baseline. Difference-in-difference (DID) analysis was used to analyze the effect of the intervention. To be exact, regression models containing time dummy (the 20-month follow-up and baseline [reference]), randomization group (intervention and control [reference]), and a time × group interaction were used to estimate the treatment effects, which allowed us to understand the impact of treatment on outcomes from baseline to the end of the follow-up period [40]. The regression model of DID is as follows:

$${Y}_{it}={\beta}_0+{\beta}_1\times {\mathrm{INTERVENTION}}_i+{\beta}_2\times {\mathrm{PERIOD}}_t+{\beta}_3\times \left({\mathrm{INTERVENTION}}_i\times {\mathrm{PERIOD}}_t\right)+{\gamma}_j{X}_{it}+{\varepsilon}_{it}$$

where *Y*_*it*_ is the outcome variable for an individual *i* in the period *t*; INTERVENTION_*i*_ is a dummy variable, which takes the value of 1 if the individual received intervention; otherwise, it is 0; PERIOD_*t*_ is the dummy variable for the period, which is 1 if the intervention is implemented, and 0 if not; the interaction term of INTERVENTION_*i*_ × PERIOD_*t*_ measures the effectiveness of the intervention, and *β*_3_ is the true effect of the intervention. *X*_*it*_ is a set of control variables, including the individual’s age, sex, household registration types, education levels, marital status, job types, life sources, medical insurance, pure-tone average, smoking status, drinking status, social activities, and social network.

Since social activities and social networks may affect the impact of the hearing rehabilitation intervention, we performed subgroup analysis by social activities and social networks to look at the effect modification. The analysis using all data was performed on the basis of “intention-to-treat (ITT).” We present the covariate-adjusted results as our main results because of the potential gains in power and precision. Stata version 16 (Stata Corporation, College Station, TX) was used for all analyses.

## Results

Table 1 shows the descriptive analysis of sociodemographic variables of 385 hearing-impaired adults aged 45 and above in the 2019 baseline hearing survey. A total of 150 adults are from the treatment group, and 235 adults are from the control group. The average age of the sample was 68.50 years old. 70.39% of the sample were males, and 93.51% were rural hukou. Nearly half of the sample (46.49%) were illiterate, and only 19.74% had secondary education or above. 76.88% of the sample were married or had a partner. Agricultural samples (49.35%) and unemployed samples (44.42%) accounted for nearly half, while non-agricultural samples only accounted for 6.23%. 85.97% of the sample received others’ support as life sources, and 96.10% of the samples had medical insurance.

Nearly 70% of the sample had severe or profound hearing loss. More than half of the sample never smoked (51.43%) or drank alcohol (52.47%). 74.03% of the sample had engaged in at least one social activity in the past month, and 73.77% of the sample had an active social network. Age, sex, hukou types, education, work type, life sources, health insurance, pure-tone average, smoking status, drinking status, social activities, and social network showed no significant differences between the treatment and control groups. The HHIE-S scores were significantly higher for those in the treatment group than those in the control group at baseline (*p*<0.001), indicating that those in the treatment group had worse hearing-related QoL. The other primary and secondary outcomes did not show significant differences between the treatment group and the control group. Overall, the characteristics of samples from baseline and follow-up surveys were well balanced between the groups.

Table 2 presents the baseline and follow-up health outcomes of hearing-impaired middle-aged and older adults by group assignment. For the primary outcomes of hearing-related QoL, the mean HHIE-S score of the baseline sample was 31.65 (SD = 0.39), of which 14.01 (SD = 0.24) for emotional problems and 17.64 (SD = 0.20) for situational problems. The mean HHIE-S score for the follow-up sample was 31.80 (SD = 0.45), of which 14.08 (SD = 0.26) for emotional problems and 17.72 (SD = 0.22) for situational problems. For the primary outcomes of generic QoL, the mean SF-12 physical standard value of the baseline sample was 40.75 (SD = 0.43). For the follow-up sample, it was 36.59 (SD = 0.62). The mean SF-12 mental standard score was 40.01 (SD = 0.49) in baseline and 45.59 (SD = 0.68) in follow-up. The mean score for baseline EQ-5D-5L was 0.76 (SD = 0.01) and for follow-up was 0.74 (SD = 0.01).

**Table 2:** Baseline and follow-up health outcomes of hearing-impaired middle-aged and older adults by group assignment

Health outcomes	Total sample (***n*** = 385)		Treatment group (***n*** = 150)		Control group (***n*** = 235)	
	Baseline	Follow-up	Baseline	Follow-up	Baseline	Follow-up
**Primary outcomes**						
HHIE-S, mean (SD)	31.65 (0.39)	31.80 (0.45)	33.49 (0.45)	31.90 (0.70)	30.47 (0.56)	31.74 (0.58)
HHIE-S-Emotional, mean (SD)	14.01 (0.24)	14.08 (0.26)	15.00 (0.30)	14.28 (0.42)	13.37 (0.34)	13.95 (0.34)
HHIE-S-Situational, mean (SD)	17.64 (0.20)	17.72 (0.22)	18.49 (0.22)	17.61 (0.34)	17.10 (0.29)	17.79 (0.29)
SF-12-Physical, mean (SD)	40.75 (0.43)	36.59 (0.62)	40.68 (0.70)	36.88 (0.99)	40.80 (0.54)	36.41 (0.79)
SF-12-Mental, mean (SD)	40.01 (0.49)	45.59 (0.68)	40.99 (0.80)	45.55 (1.10)	39.39 (0.62)	45.62 (0.88)
EQ-5D-5L, mean (SD)	0.76 (0.01)	0.74 (0.01)	0.75 (0.02)	0.74 (0.02)	0.77 (0.01)	0.74 (0.02)
**Secondary outcomes**						
Chronic diseases, N (%)						
No	101 (26.23)	90 (25.86)	46 (30.67)	36 (27.07)	55 (23.40)	54 (25.12)
Yes	284 (73.77)	258 (74.14)	104 (69.33)	97 (72.93)	180 (76.60)	161 (74.88)
ADLs, *N* (%)						
Unimpaired	295 (76.62)	311 (89.37)	119 (79.33)	120 (90.23)	176 (74.89)	191 (88.84)
Impaired	90 (23.38)	37 (10.63)	31 (20.67)	13 (9.77)	59 (25.11)	24 (11.16)
IADLs, *N* (%)						
Unimpaired	123 (31.95)	179 (51.14)	40 (26.67)	70 (51.85)	83 (35.32)	109 (50.70)
Impaired	262 (68.05)	171 (48.86)	110 (73.33)	65 (48.15)	152 (64.68)	106 (49.30)
Depressive symptoms, *N* (%)						
No	245 (63.64)	196 (56.00)	92 (61.33)	79 (58.52)	153 (65.11)	117 (54.42)
Yes	140 (36.36)	154 (44.00)	58 (38.67)	56 (41.48)	82 (34.89)	98 (45.58)
Cognition scores measured by MMSE, mean (SD)	16.93 (0.45)	17.40 (0.46)	17.07 (0.75)	17.22 (0.72)	16.83 (0.56)	17.51 (0.59)

*Abbreviations*: *HHIE-S* Hearing Handicap Inventory for the Elderly Screening Version, *SF-12* 12-Item Short-Form Health Survey, *EQ-5D-5L* EuroQoL 5-Dimension 5-Level, *ADLs* activities of daily living, *IADLs* instrumental activities of daily living, *MMSE* Mini-Mental State Exam

For secondary outcomes, physical health indicators included chronic diseases, ADLs, and IADLs, and the mental health indicator included depressive symptoms. 26.23% of the baseline sample and 25.86% of the follow-up sample reported having at least one chronic disease. The proportion of people with impaired ADLs in baseline and follow-up was 23.38% and 10.63%, respectively. The percentage of people with impaired IADLs in baseline was 68.05% and in follow-up was 48.86%. 36.36% of the baseline sample and 44.00% of the follow-up sample had depressive symptoms, while in the follow-up, a higher percentage of 41.48% in the treatment group and 45.58% in the control group had depressive symptoms. The mean cognitive scores were relatively low at 16.93 (SD = 0.45) for the baseline sample and 17.40 (SD = 0.46) for the follow-up sample, as most of the respondents were poorly educated (illiterate or just finishing primary school) and had great difficulty hearing.

Table 3 shows the effects of the hearing aid rehabilitation intervention on health outcomes using the DID method. For the SF-12 physical and mental value, the EQ-5D-5L value, and the cognitive score, a positive coefficient indicated the intervention led to good health status. For the HHIE-S score, chronic diseases, ADLs, IADLs, and depressive symptoms, a negative coefficient indicated that the intervention led to good health. For primary outcomes, although those in the treatment group had worse hearing-related QoL at baseline, the hearing aid rehabilitation intervention significantly improved hearing-related QoL (interaction coefficient for HHIE-S = − 2.86, *p* = 0.005), hearing-related emotional QoL (interaction coefficient for HHIE-S-Emotional = − 1.42, *p* = 0.029), and hearing-related situational QoL (interaction coefficient for HHIE-S-Situational = − 1.43, *p* = 0.007). For secondary outcomes, the intervention significantly alleviated the increase in depressive symptoms (interaction term = − 0.14, *p* = 0.042).

**Table 3:** Differences in health outcomes between the intervention and control groups during baseline and follow-up hearing surveys

Health outcomes	DID		
	Treatment × time coefficient	95% CI	***p*** value
**Primary outcomes**			
HHIE-S	− 2.86	− 4.85, − 0.86	0.005
HHIE-S-Emotional	− 1.42	− 2.70, − 0.15	0.029
HHIE-S-Situational	− 1.43	− 2.47, − 0.40	0.007
SF-12-Physical	0.67	− 2.86, 4.20	0.711
SF-12-Mental	− 2.23	− 5.93, 1.47	0.236
EQ-5D-5L	0.02	− 0.04, 0.08	0.583
**Secondary outcomes**			
Chronic diseases	0.05	− 0.07, 0.17	0.426
ADLs	0.03	− 0.09, 0.14	0.648
IADLs	− 0.09	− 0.23, 0.04	0.178
Depressive symptoms	− 0.14	− 0.27, − 0.01	0.042
Cognition scores	0.08	− 1.76, 1.91	0.935

All models adjusted for age, sex, hukou types, education, marital status, work types, life sources, health insurance, pure-tone average, smoking status, drinking status, social activities, and social network

*Abbreviations*: *HHIE-S* Hearing Handicap Inventory for the Elderly Screening Version, *SF-12* 12-Item Short-Form Health Survey, *EQ-5D-5L* EuroQoL 5-Dimension 5-Level, *ADLs* activities of daily living, *IADLs* instrumental activities of daily living

Table 4 presents a subgroup analysis of DID by social activities and social network, so as to analyze the differences in health outcomes between intervention and control groups. The hearing aid rehabilitation intervention did not improve health in individuals with no social activities or with restricted social networks. For individuals who had social activities in the past month, the hearing aid rehabilitation intervention significantly improved hearing-related QoL (interaction coefficient for HHIE = − 3.89, *p* = 0.001), hearing-related emotional QoL (interaction coefficient for HHIE-Emotional = − 2.08, *P* = 0.007), and hearing-related situational QoL (interaction coefficient for HHIE-Situational = − 1.81, *p* = 0.005). For individuals who had active social network, the hearing aid intervention significantly improved hearing-related QoL (interaction coefficient for HHIE = − 2.80, *p* = 0.016) and hearing-related situational QoL (interaction coefficient for HHIE-Situational = − 2.76, *p* = 0.009) and significantly alleviated the increase in depressive symptoms (interaction term = − 0.16, *p* = 0.045).

**Table 4:** Differences in health outcomes between the intervention and control groups during baseline and follow-up hearing surveys, by social activities and social network

**Health outcomes**	**No social activities**^**a**^			**Have social activities**^**a**^		
	**Treatment × time coefficient**	**95% CI**	***p*** **value**	**Treatment × time coefficient**	**95% CI**	***p*** **value**
HHIE-S	0.35	− 3.62, 4.31	0.862	− 3.89	− 6.27, − 1.52	0.001
HHIE-S-Emotional	0.51	− 2.03, 3.05	0.691	− 2.08	− 3.58, − 0.58	0.007
HHIE-S-Situational	− 0.16	− 2.15, 1.83	0.871	− 1.81	− 3.07, − 0.55	0.005
Depressive symptoms	− 0.14	− 0.44, 0.16	0.357	− 0.12	− 0.28, 0.03	0.109
	**Restricted social network**^**b**^			**Active social network**^**b**^		
HHIE-S	− 2.97	− 7.47, 1.54	0.194	− 2.80	− 5.08, − 0.52	0.016
HHIE-S-Emotional	− 2.10	− 4.90, 0.70	0.139	− 1.22	− 2.70, 0.25	0.103
HHIE-S-Situational	− 0.86	− 3.08, 1.35	0.440	− 1.58	− 2.76, − 0.39	0.009
Depressive symptoms	− 0.07	− 0.34, 0.20	0.612	− 0.16	− 0.31, − 0.01	0.045

*Abbreviations*: *HHIE-S* Hearing Handicap Inventory for the Elderly Screening Version

^a^All models adjusted for age, sex, hukou types, education, marital status, work types, life sources, health insurance, pure-tone average, smoking status, drinking status, and social activities

^b^All models adjusted for age, sex, hukou types, education, marital status, work types, life sources, health insurance, pure-tone average, smoking status, drinking status, and social network

## Discussion

Our RCT is one of the first rigorously designed trials on the effects of providing free hearing aids on multiple health outcomes in developing countries. The results showed that the intervention was to some extent effective in improving hearing-related QoL but had no effects on generic QoL. The intervention also alleviated the increase in depressive symptoms in the 20-month follow-up for middle-aged and older adults in rural China. The subgroup analysis revealed that the effects were only shown among people with social activities or active social networks. That is, the intervention was effective for those who were socially active. As most previous trials that have attempted to improve health by wearing hearing aids have been done in high-income countries, our study provides a promising vision of hearing aid fitting strategies for developing countries.

Although the results were statistically significant, the HHIE-S score of the treatment group only improved a little from baseline to follow-up. However, we should also account for the fact that the HHIE-S score of the control group worsened from baseline to follow-up, making the total effect size larger. In addition, it appears that participants in the treatment arm actually had more depressive symptoms at follow-up than they did at baseline, which was possibly due to older age or the COVID-19. The intervention actually alleviated the increase in depressive symptoms in that the increase in depressive symptoms was not as large in the treatment group as in the control group. Our results were consistent with existing studies. For example, one pioneering study found that, compared with those in the control group, the social emotional function and communication ability measured by the HHIE of older adults with hearing loss were significantly improved, and their depressive symptoms were significantly reduced after wearing hearing aids [41].

However, there were no effects of the hearing aid intervention on generic QoL, which also conformed to the previous literature. For example, Jorre et al. found no significant improvements in mobility, self-care, daily activities, and pain/discomfort in the five dimensions of QoL measured by EQ-5D after wearing hearing aids [17, 18]. Stark et al. revealed that hearing aid use did not significantly improve physical function, general health, and mental health of QoL measured by SF-36. Vuorialho et al. also suggested that the QoL as measured by EQ-5D in hearing-impaired people did not improve after wearing hearing aids [19]. The reasons may be that, comprehensive indicators of QoL like SF-36, EQ-5D often ignore the evaluation of communication problems or are rarely affected by the psychological, social, and emotional outcomes of those hearing-impaired [42], so these scales can be lack of sensitivity when applying to related studies on hearing loss [18].

Heterogeneity analysis found that the effects of the hearing aid intervention on health outcomes varied by social activities and social networks across people. For adults with no social activities or with restricted social networks, the hearing aid intervention did not improve their health. For adults participating in at least one social activity, the hearing aid intervention significantly improved hearing-related QoL and its emotional and situational dimension. For adults with active social networks, the hearing aid intervention not only improved hearing-related QoL, but also significantly alleviated the increase in depressive symptoms. Hearing loss has been thought to be associated with reduced social interaction and emotional communication [43]. It can be speculated that individuals participating in social activities and with active social networks may have a stronger desire to interact and communicate with others, and hearing aids greatly reduce communication barriers, which is conducive to the improvement of health conditions [44].

Our study is the first RCT in China to measure the effects of the hearing aid intervention and its heterogeneity in hearing-impaired middle-aged and older adults. Strengths of the study include its randomized controlled design, a large sample size, and high (> 85%) rates of follow-up, which tend to increase confidence in the significance of the results. However, there are also several limitations. First, as the follow-up data were collected during the COVID-19 pandemic, the related restrictions on socialization and mental well-being may potentially reduce the effect sizes. Second, given that most individuals had severe to profound hearing loss and cognitive impairment, we should be cautious when generalizing the results to a larger population. Fourth, our RCT with an open hearing aid is a limitation, as expectancy-based placebo effects can affect the outcomes prone to expectancy results, such as the self-reported hearing-related measure and the depression measure. Future studies could take into account other factors like the pandemic or hearing aid compliance and generalize the results to a broader population.

## Conclusion

To conclude, our study proves that proving free hearing aids substantially improved hearing-related QoL and alleviated the increase in depressive symptoms for middle-aged and older adults with hearing loss in rural China. It is imperative that middle and older adults with age-related hearing loss wear hearing aids regularly to recover their hearing, as well as strengthen their social participation and social network to further improve QoL. The intervention is expected to be scaled up through its integration within the health insurance system in China and be adapted to other similar settings in developing countries facing the problem of untreated hearing loss.

## Data Availability

The datasets used and/or analyzed during the current study are available from the corresponding author on reasonable request.

## Abbreviations

CI: Confidence interval
CVD: Cardiovascular disease
HbA1c: Glycated hemoglobin
HR: Hazard ratio
NHANES III: Third National Health and Nutrition Examination Survey
NOS: Newcastle-Ottawa Scale
OR: Odds ratio
PRISMA: Preferred Reporting Items for Systematic Reviews and Meta-Analyses
RR: Relative risk
T2DM: Type 2 diabetes mellitus (T2DM)
T3: Triiodothyronine
T4: Thyroxine
TSH: Thyroid-stimulating hormone
